# BCG and Adverse Events in the Context of Leprosy

**DOI:** 10.3389/fimmu.2018.00629

**Published:** 2018-04-04

**Authors:** Renate Richardus, Anouk van Hooij, Susan J. F. van den Eeden, Louis Wilson, Korshed Alam, Jan Hendrik Richardus, Annemieke Geluk

**Affiliations:** ^1^Department of Infectious Diseases, Leiden University Medical Center, Leiden, Netherlands; ^2^Department of Public Health, Erasmus MC, University Medical Center Rotterdam, Rotterdam, Netherlands; ^3^Rural Health Program, The Leprosy Mission International Bangladesh, Nilphamari, Bangladesh

**Keywords:** adverse events, BCG (re)vaccination, biomarker profiles, household contacts, protective immunity, leprosy, *Mycobacterium leprae*

## Abstract

**Background:**

Notwithstanding its beneficial immunoprophylactic outcomes regarding leprosy and childhood TB, BCG vaccination may cause adverse events, particularly of the skin. However, this local hyper-immune reactivity cannot be predicted before vaccination, nor is its association with protection against leprosy known. In this study we investigated the occurrence of adverse events after BCG (re)vaccination in contacts of leprosy patients and analyzed whether the concomitant systemic anti-mycobacterial immunity was associated with these skin manifestations.

**Methods:**

Within a randomized controlled BCG vaccination trial in Bangladesh, 14,828 contacts of newly diagnosed leprosy patients received BCG vaccination between 2012 and 2017 and were examined for adverse events 8 to 12 weeks post-vaccination. From a selection of vaccinated contacts, venous blood was obtained at follow-up examination and stimulated with *Mycobacterium leprae* (*M. leprae*) antigens in overnight whole-blood assays (WBA). *M. leprae* phenolic glycolipid-I-specific antibodies and 32 cytokines were determined in WBAs of 13 individuals with and 13 individuals without adverse events after vaccination.

**Results:**

Out of the 14,828 contacts who received BCG vaccination, 50 (0.34%) presented with adverse events, mainly (80%) consisting of skin ulcers. Based on the presence of BCG scars, 30 of these contacts (60%) had received BCG in this study as a booster vaccination. Similar to the pathological T-cell immunity observed for tuberculoid leprosy patients, contacts with adverse events at the site of BCG vaccination showed elevated IFN-γ levels in response to *M. leprae*-specific proteins in WBA. However, decreased levels of sCD40L in serum and GRO (CXCL1) in response to *M. leprae* simultaneously indicated less T-cell regulation in these individuals, potentially causing uncontrolled T-cell immunity damaging the skin.

**Conclusion:**

Skin complications after BCG vaccination present surrogate markers for protective immunity against leprosy, but also indicate a higher risk of developing tuberculoid leprosy.

**Clinical Trial Registration:**

Netherlands Trial Register: NTR3087.

## Introduction

Despite effective treatment of leprosy patients with multidrug therapy (MDT), the global number of new cases has not declined during the past decennium (1). A plausible explanation for this *status quo* could be that contacts of leprosy patients are prolonged and repetitively exposed to *Mycobacterium leprae* (*M. leprae*) before treatment of index cases is initiated, leading to continued bacterial transmission. Therefore, new tools and methodologies, such as immuno- and chemoprophylaxis regimens, are needed to interrupt transmission.

BCG vaccination offers variable protection against tuberculosis (2) and other mycobacterial diseases such as leprosy (3) and Buruli ulcer (4). Moreover, recently it has become clear that BCG can modulate the innate immune system also leading to protection through a mechanism referred to as trained immunity (5–7). The protective effect against TB thus induced in children by neonatal BCG vaccination influences cytokine responses to heterologous pathogens, an effect that is reported to be characterized by decreased anti-inflammatory cytokine responses, but increased IL-6 (5, 8).

In a previous study, immunoprophylaxis by BCG vaccination of contacts of newly diagnosed leprosy patients in Bangladesh conferred 56% protection, but was not affected by previous childhood BCG vaccination (9).

Although chemoprophylaxis does not protect a given individual from subsequent exposure to bacilli, the use of a single-dose rifampicin (SDR) in contacts in that study showed prevention of 56% in the first 2 years after chemoprophylaxis and treatment of the index case (10). Strikingly, if contacts had received BCG vaccination as part of a childhood vaccination program (as determined by the presence of a BCG-scar), the protective effect of SDR even reached 80%.

To investigate whether the effects of SDR and BCG can be complimentary, a cluster randomized controlled BCG vaccination trial is currently conducted in Bangladesh, analyzing the potential synergetic effect of these chemo- and immunoprophylactics by comparing the effect of BCG vaccination alone versus BCG followed by SDR after 8 to 12 weeks to prevent leprosy in contacts of new leprosy cases (designated the MALTALEP trial) (11).

In Bangladesh, BCG is routinely given to infants as part of the neonatal vaccination scheme as a prophylactic vaccine against tuberculosis. The coverage of BCG vaccination is estimated to be 98%^1^. Based on the visibility of BCG vaccination scars, 8,430 out of 14,779 contacts (57%) within this trial had received BCG vaccination at birth. However, since not all individuals receiving BCG develop a visible scar (12), this number is probably higher.

BCG vaccination has been reported to cause adverse effects within BCG childhood vaccination programs in endemic areas (13–16) as well as in BCG naïve individuals in leprosy and TB non-endemic areas (17–20). In the current study, we investigated the number and nature of adverse events occurring after BCG vaccination in the MALTALEP trial.

In addition, to investigate whether these adverse events can provide further insight into the protective effect of BCG, we analyzed cytokine production in *M. leprae*-antigen-stimulated whole-blood assays (WBA) of 13 contacts developing adverse events and 13 contacts matched for age and gender, lacking such complications.

## Materials and Methods

### Study Population

Newly diagnosed leprosy patients and their household contacts (HCs) were recruited on a voluntary basis between 2012 and 2017 (Table 1). Leprosy was diagnosed based on clinical and bacteriological analysis and classified according to Ridley and Jopling (21). Leprosy patients were treated according to WHO standards. Contacts of consecutively diagnosed new leprosy patients were included in the districts of Nilphamari, Rangpur, Thakurgaon, and Panchagarh, in the northwest of Bangladesh (11). Each contact group consisted of around 15 contacts and were randomly assigned to receive BCG or BCG plus rifampicin. Immunization with BCG was given to all included contacts, when the index case received the second dose of MDT. At intake, before BCG vaccination, all contacts were examined for a BCG scar on the left upper arm. After 8 to 12 weeks, vaccinated contacts were reviewed for adverse events during follow-up examination. Contacts were categorized as household members (sharing either roof, kitchen or both) or direct neighbors. Contacts were excluded from the study according to criteria described previously.

**Table 1:** Characteristics of contacts with or without complication after BCG vaccination.

	Contacts with complication after BCG(% of total)	Contacts without complication after BCG	Total contacts who received BCG	*p*-value
Contacts	50	14,778	14,828	n.a.
Male	23 (0.34%)	6,677	6,700	0.91
Female	27 (0.33%)	8,101	8,128	
Child (5–16 years)	21 (0.43%)	4,829	4,850	0.16
Adult	29 (0.29%)	9,949	9,978	
No BCG scar visible	20 (0.32%)	6,336	6,356	0.68
BCG scar present	30 (0.35%)	8,430	8,460	
Vaccination status unknown	0	12	12	n.a.
Index with MB	19^a^ (4.08%)	447	466	0.08
Index with PB	26^b^ (2.42%)	1,047	1,073	

*^a^One household with a multibacillary (MB) index had two contacts with a BCG complication*.

*^b^One household with a paucibacillary (PB) index case had two contacts with a BCG complication, another household even had four contacts with a BCG complication*.

### Leprosy Prevalence

During this study, the prevalence in the four districts (Nilphamari, Rangpur, Panchagarh and Thakurgaon) in the northwest of Bangladesh was 0.82 per 10,000 with a new case detection rate of 0.98 per 10,000 (monthly report of the Rural Health Program of these four districts).

### Ethics

The MALTALEP trial is performed according to standard Good Clinical Practice (GCP) guidelines.^2^ Participants were informed about the study objectives, the samples, and their right to refuse to take part in or withdraw from the study without consequences for their treatment. Written informed consent was obtained before enrollment from all participants. For illiterate people a thumb print was taken, and for minors under 16 years of age, the guardian’s additional consent was obtained. All patients received treatment according to national guidelines. Participants were informed about the potential adverse events of the trial, that free consultation and treatment would be offered in case of adverse events and requested to report any suspected adverse events to the responsible field worker. Ethical approval of the study-protocol was obtained through the National Research Ethics Committee (Bangladesh Medical Research Council; Protocol no. BMRC/NREC/2010-2013/1534).

### BCG Vaccination

Vaccination was performed between September 2012 and February 2017. BCG was administered intradermally. The BCG vaccine used in this trial (Japan BCG Laboratory, Tokyo, Japan) is also used in the routine neonatal vaccination program of Bangladesh. Vaccines were stored at the State Immunization Program facilities in the four different districts of the study area and kept at 0–4°C throughout the fieldwork.

### Adverse Events

All contacts receiving vaccination were provided with a vaccination card with details on how to reach the researcher in case of questions or adverse events. Contacts with self-reported adverse events were examined by field staff. Additionally, all contacts were examined 8 to 12 weeks after administration of the BCG. Data on adverse events were collected on the MALTALEP Contact Registration forms and on a separate BCG complication form (11). In the case of an adverse event following BCG complication, contacts were referred to the state tuberculosis medical officers for treatment. Ulcers were considered abnormal if they were larger than 10 mm diameter in size, or if they presented in combination with fever and malaise. Contacts were also checked for the presence of lymphadenopathy, abnormal scarring and keloids and if the course of the complication was different than normal. To document the size of the ulcers, pictures were taken of each BCG complication case and stored in a database.

### Samples for Immunological Analysis

Blood was drawn from 15 contacts who developed an adverse event after receiving BCG vaccination. Two contacts were excluded from the analysis, because they later developed leprosy. Cytokine levels in WBA of 13 contacts with adverse events were analyzed and compared with those in contacts without (a scar or ulcer of <10 mm). WBA were performed for both groups and anti-phenolic glycolipid-I (PGL-I) serology cytokines and chemokines concentrations in supernatants were assessed.

### Whole Blood Assays (WBA)

Venous blood was drawn from contacts at the time BCG complications occurred, which was on average 7.9 weeks after receiving BCG. As a control group, contacts without complications were tested. Controls were matched for age and gender as well as time point at which blood was drawn (on average 7.7 weeks; Table 2). Heparinized blood (4 mL) was directly added to microtubes pre-coated with *M. leprae* whole cell sonicate (WCS), *M. leprae*-unique recombinant proteins ML2478 and ML0840 (designated Mlep) (22), or without antigen stimulus (designated NIL) (11, 23). After 24-h incubation at 37°C materials were frozen at −20°C, shipped on dry ice to the LUMC, and stored at −80°C until analysis.

**Table 2:** Characteristics of contacts with BCG-related complications and matched controls.

	Complications	No complications
Number of contacts	13	13
Average age (years)	33.8	36.2
Number of females	8	8
Number of males	5	5
Average no. of weeks between BCG and WBA	7.9 (1.0–13.5)	7.7 (4.0–10.0)
Presence of BCG scar before study	8	6
Average size of BCG scar/ulcer (in mm)	14.8 (4.5–27)	3.4 (2.5–4.5)
Received SDR before blood drawing	1^b^	3^a^
Received no SDR	12	10

*^a^All controls received SDR 2 weeks before blood was drawn*.

*^b^The contacts with a complication after BCG vaccination received SDR 4 weeks before experiencing the adverse event at 13 weeks post vaccination*.

### Cytokine-Chemokine Analysis

sCD40L, EGF, G-CSF, GM-CSF, GRO, IFN-α2, IFN-γ, IL-1α, IL-1β, IL-1ra, IL-2, IL-4, IL-6, IL-8, IL-10, IL-12(p40), IL-12(p70), IL-17A, IP-10, MCP-1, MCP-3, MDC (CCL22), MIP-1α, MIP-1β, PDGF-AB/BB, PDGF-AA, RANTES, TGF-α, TNF-α, TNF-β, VEGF, and Eotaxin (CCL11) in WBA supernatants were measured with the Milliplex magnetic bead kit (Merck, USA) on 96 well multiscreen filter plates (Millipore, USA) using the Bio-Plex-100-suspension-array-system (BioRad, Veenendaal) and analyzed using the Bio-Plex Manager software 6.1 (Bio-Rad Laboratories, Veenendaal, The Netherlands) (22). After pre-wetting the filter with assay solution, supernatant samples (25 µL) were added to the plates, together with 25-µL assay buffer and 25-µL beads, and the plates were incubated overnight at 4°C. After two washing steps with 200-µL wash buffer using a vacuum pump (Millipore, USA), 25-µL detection Ab mixture was added per well, and plates were incubated at room temperature in the dark for 1 h on a plate shaker at 300 rpm. Streptavidin-PE solution (25 µL per well) was added and incubated for 30 min at room temperature in the dark. After two washes, 150-µL Sheath Fluid was added to each well, and the plates were placed in the Bio-Plex System. From each well, a minimum of 50 analyte-specific beads were analyzed for fluorescence. A curve fit was applied to each standard curve according to the manufacturer’s manual. Sample concentrations were interpolated from these standard curves. Analyte concentrations outside the upper or lower limits of quantification were assigned the values of the limits of quantification of the cytokine or chemokine.

### PGL-I and *M. leprae* WCS

Synthesized disaccharide epitope [3,6-di-O-methyl-β-D-glucopyranosyl(1→4)2,3-di-O-methylrhamnopyranoside], similar to *M. leprae*-specific PGL-I glycolipid, coupled to human serum albumin (synthetic PGL-I; designated ND–O–HSA) and *M. leprae* WCS, generated with support from the NIH/NIAID Leprosy Contract N01-AI-25469, were obtained through the Biodefense and Emerging Infections Research Resources Repository^3^ (24).

### PGL-I ELISA

IgM and IgG antibodies against synthetic PGL-I were detected as previously described adapted for the use of specific IgM and IgG antibody detection (22, 25). A synthetic analog of the *M. leprae*-specific PGL-I (ND–O–HSA) was coated onto high-affinity polysorp Immulon 4HBX 96-well Nunc ELISA plates (Thermo Scientific, Rochester, NY, USA) using 200 ng/well in 50-µL 0.1-M sodium carbonate/bicarbonate pH 9.6 (i.e., coating buffer) at 4°C overnight. Unbound Ag was removed by washing with PBS containing 0.05% Tween 20 (washing buffer) six times and wells were blocked with PBS containing 1% BSA (Roche Diagnostics, Germany) and 0.05% Tween 80 for 1 h at room temperature. 50 µL of 1:400 diluted serum/plasma (PBS/0.01% BSA as dilution buffer) was added to the wells and incubated for 2 h at room temperature. After incubation, wells were washed six times with washing buffer, followed by the addition of 50 µL of 1:8,000 antihuman IgM-HRP (Sigma A6907) or 1:4,000 antihuman IgG-HRP (DAKO P0214) and incubated for 2 h at room temperature. Following washing, the wells with the wash buffer, 50 µL 3.3′,5.5′-tetramethylbenzidine (TMB) was added and the color reaction was stopped using H2SO4 after 10–15 min. The absorbance was determined at wavelength of 450 nm. Samples with an optical density (OD_450_), after correction for background, >0.20 were considered positive. The cutoff for positivity was determined by a threefold multiplication of the average value for non-endemic control individuals.

### Statistical Analysis

Statistical analysis was performed using GraphPad Prism version 7 (GraphPad Software, San Diego, CA, USA),^4^ SPSS Statistics 24,^5^ and R Version 3.3.0 (R, Vienna, Austria).^6^ A chi-square test was performed for contacts who developed BCG complications to identify potential differences compared with the control contacts’ characteristics (Table 1). A significance level of *p* ≤ 0.05 was used.

For identification of an immune biomarker signature associated with skin complications after BCG vaccination, a global test was used (26), which provided hierarchical clustering of the cytokines/chemokines based on absolute correlation difference and average linkage. Moreover, the Mann–Whitney *U* test was performed to identify differences in group mean levels of host markers. The statistical significance level used was *p* ≤ 0.05. For significantly different markers in both the global test and Mann–Whitney *U* test, the diagnostic potential was assessed by receiver operating characteristic curve (ROC) analysis to determine the area under the curve (AUC). The cutoff values for optimal sensitivity and specificity were determined by calculating the Youden’s Index (27). To construct a biomarker profile, a linear discriminant analysis (LDA) was performed in SPSS. Analytes were ranked based on the pooled within-group correlations between discriminating variables and standardized canonical discriminant functions. The six most contributing analytes to the discriminant function were selected to construct a biomarker profile. The profile was constructed stepwise, determining the optimal sensitivity and specificity for each step. The optimal cutoff was determined per analyte after which each individual was designated positive or negative for all analytes separately.

## Results

### Occurrence of Adverse Events After BCG Vaccination

**Figure 1:**
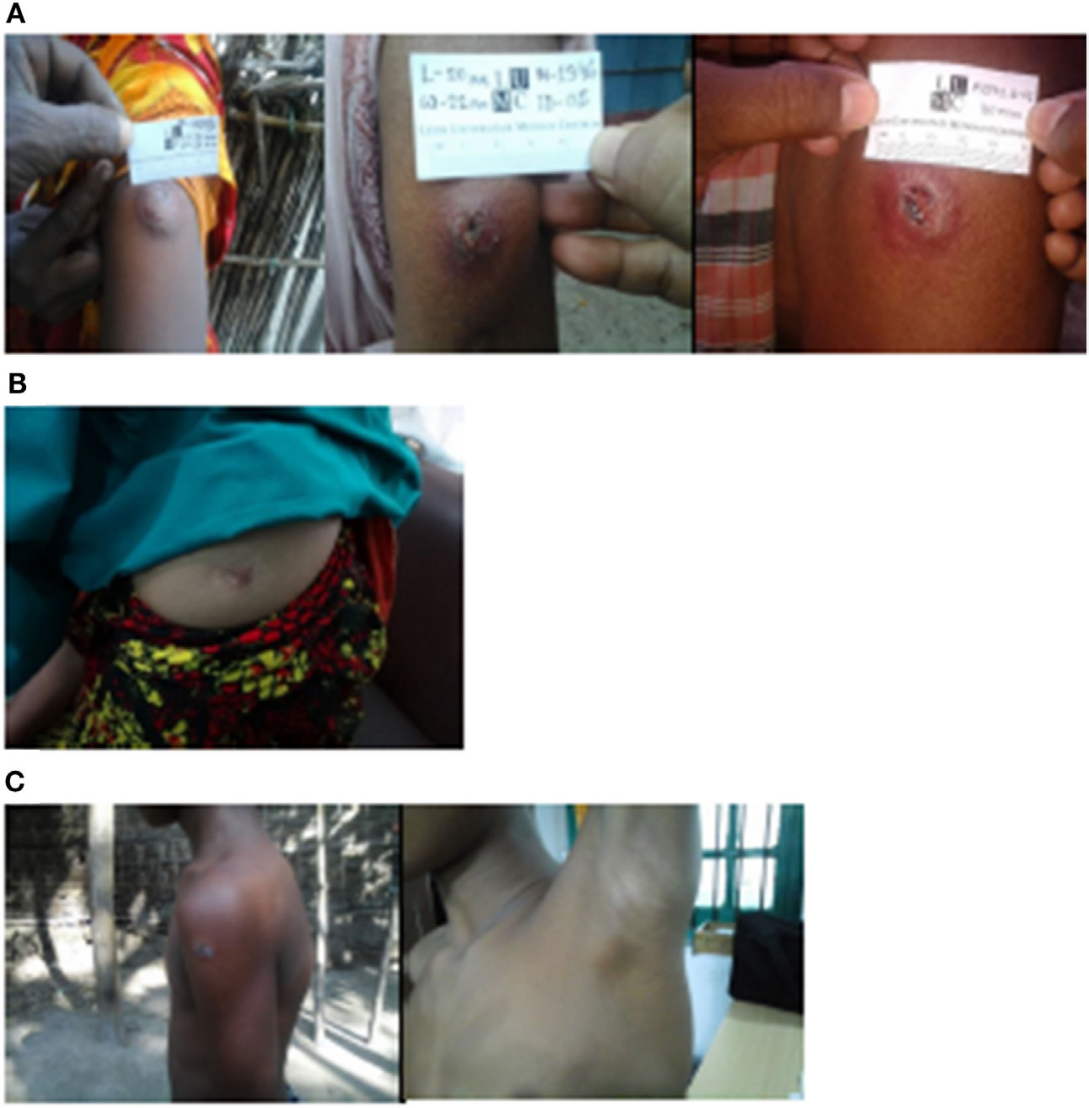
Representative examples of skin complications after BCG vaccination. **(A)** Three contacts with big ulcers (>10 mm). **(B)** A contact with keloid (picture taken before operation). **(C)** A contact with an ulcer and lymphadenitis who developed leprosy at follow-up.

Out of the 14,828 contacts who received BCG vaccination within the trial, 50 (0.34%) presented with vaccination-related adverse events (Table 1). The most common adverse events were skin ulcers (Table S1 in Supplementary Material; Figure 1A). A total of 40 contacts (80%) developed large skin ulcers varying between 10 and 35 mm; four of these also had axillary lymphadenopathy and one had enlarged lymph nodes. One ulcer was 8 mm, but was included as adverse event because the contact also reported malaise and mild fever. Keloids (Figure 1B) were present in eight contacts, of whom three were small (<1 cm) and three were >1 cm. One contact developed a persistent keloid, which was first signaled 1 year after receiving BCG vaccination. When excluding the contact with persistent keloid, the average time between BCG vaccination and initiation of complication in the 50 contacts was 5.5 weeks.

### Variations in BCG-Vaccination-Related Adverse Events

In four contacts, adverse events manifested differently: one woman developed an abscess, which was incised and drained at home 3 months after vaccination, then developed intermittent fever and was treated unsuccessfully with various antibiotics of unknown kind provided by different doctors. After 1 year, the contact was admitted for investigation, because of an erythematous nodule (2 cm × 2 cm) surrounded by scarring. She was re-incised by a plastic surgeon upon suspicion of a deep-seated abscess. The histological report of the biopsy showed a keloid scar (Figure 1B).

A second contact had a persistent pustule of 5 mm 5 months after receiving BCG, felt weak, and had coughed for the past 2 months. She only had a 2-day history of fever and was tested sputum-negative for acid-fast bacilli (AFB). The pustule was not opened, but kept clean and dry and healed after a course of flucloxacillin.

A third contact had developed a large scar (12 mm × 10 mm) and many small ulcers on both arms and legs after receiving BCG. She received unknown medication from an outside doctor and the lesions healed.

Finally, the fourth contact presented with an ulcer at the BCG injection site of 10 mm × 15 mm and mild left axillary lymphadenopathy. Already before BCG vaccination, the contact had a history of occasional fever and pain palpable on the ribs, which was treated with pain killers. He had no known contact to TB patients, and was sputum- and X-ray negative for TB.

In addition to adverse events, two contacts also developed leprosy following BCG vaccination (Figure 1C). One had a small keloid, and the other had an ulcer of 15 mm × 20 mm with lymphadenitis (Figure 1B).

The average age at the time of the adverse event was 30 years, with a range of 6 to 80 years. Similar numbers of males and females were identified with adverse events (Table 1). More than half (60%) received a revaccination, based on the presence of a BCG scar. A higher number of children aged between 5 and 16 years old (as aged under 5s were excluded) developed BCG adverse events compared with adults (0.43% versus 0.29%); however, this number was not statistically significant (*p* = 0.16; Table 1). A slightly higher but statistically not significant number of contacts who received BCG for the second time developed adverse events compared with those who lacked a BCG scar (0.35% versus 0.32%; *p* = 0.68). Despite that an almost double amount of contacts developed adverse events when the index patient had multibacillary (MB) leprosy, compared with paucibacillary (PB) leprosy, this increase was not statistically significant either (*p* = 0.08).

Among the 13 contacts with an adverse event after BCG from whom blood was analyzed, nine had large ulcers >10 mm, one patient had an ulcer of 8 mm, but with general malaise, one had a keloid, one had a big scar, and one had an enlarged lymph node.

### Anti-PGL-I IgM Levels

**Figure 2:**
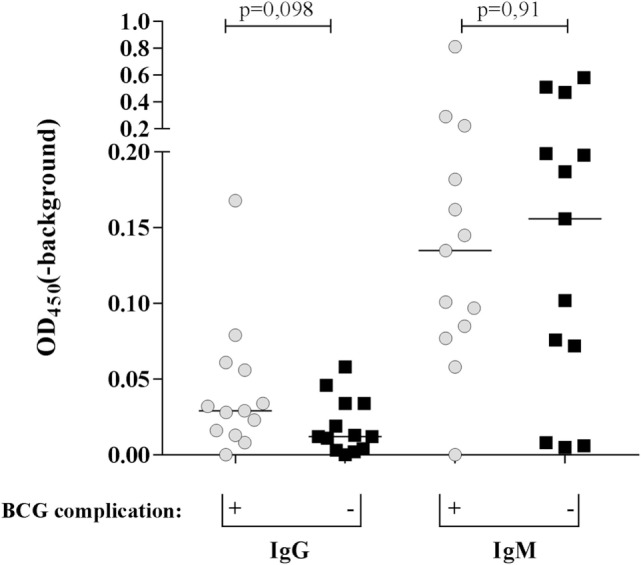
*Mycobacterium leprae* phenolic glycolipid-I (PGL-I)-specific antibodies in contacts of leprosy patients with or without BCG-induced skin complications. IgG and IgM antibodies directed against synthetic PGL-I (ND–O–HSA) were determined by ELISA. Samples with OD_450_ (corrected for background OD) >0.2 were considered seropositive. No statistically different levels of IgG and IgM antibodies were observed between the contacts with (+ ; gray dots) or without (−; black squares) complications.

To estimate whether the extent of seropositivity in contacts of leprosy patients could already indicate whether complications could occur after BCG vaccination, the levels of anti-*M. leprae* PGL-I IgM antibodies, as estimated by the optical density at 450 nm (OD_450_), were measured in sera of 26 individuals: 13 with and 13 without BCG complications (Figure 2). Three contacts from both groups were seropositive for IgM against PGL-I (OD_450_ > 0.2), but no significant differences were observed between both groups.

### Immune Profiles Coinciding With Adverse Events After BCG Vaccination

**Figure 3:**
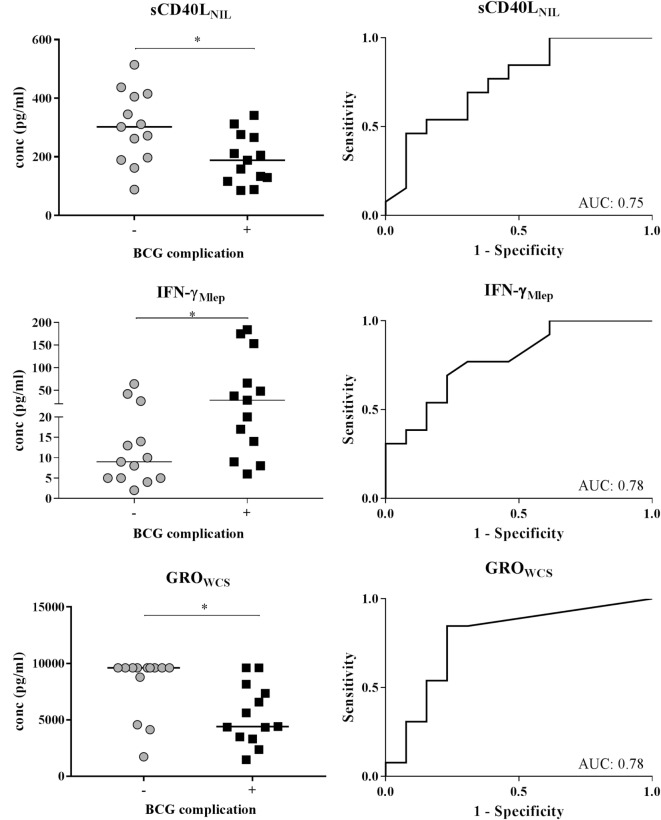
Cytokine concentrations in 24-h whole-blood assays (WBA) with or without stimulation with *Mycobacterium leprae* (*M. leprae*) unique proteins (Mlep) or *M. leprae* whole cell sonicate (WCS) in contacts with and without BCG complications (left panels). The global test (26) indicated that sCD40Lmed, GROwcs, and IFN-γ_Mlep_ were significantly different between BCG-vaccinated contacts of leprosy patients with BCG-related complications and those without. This was confirmed by a Mann–Whitney *U* test. *p < 0.05–0.01. Receiver operating characteristic curves (ROCs) were computed and the area under the curve (AUC) is indicated for each analyte (right panels). The limits of detections for sCD40Lmed were 1.5–10,000, for GROwcs were 12.5–9,600. and IFN-γ_Mlep_ were 2–10,000.

**Figure 4:**
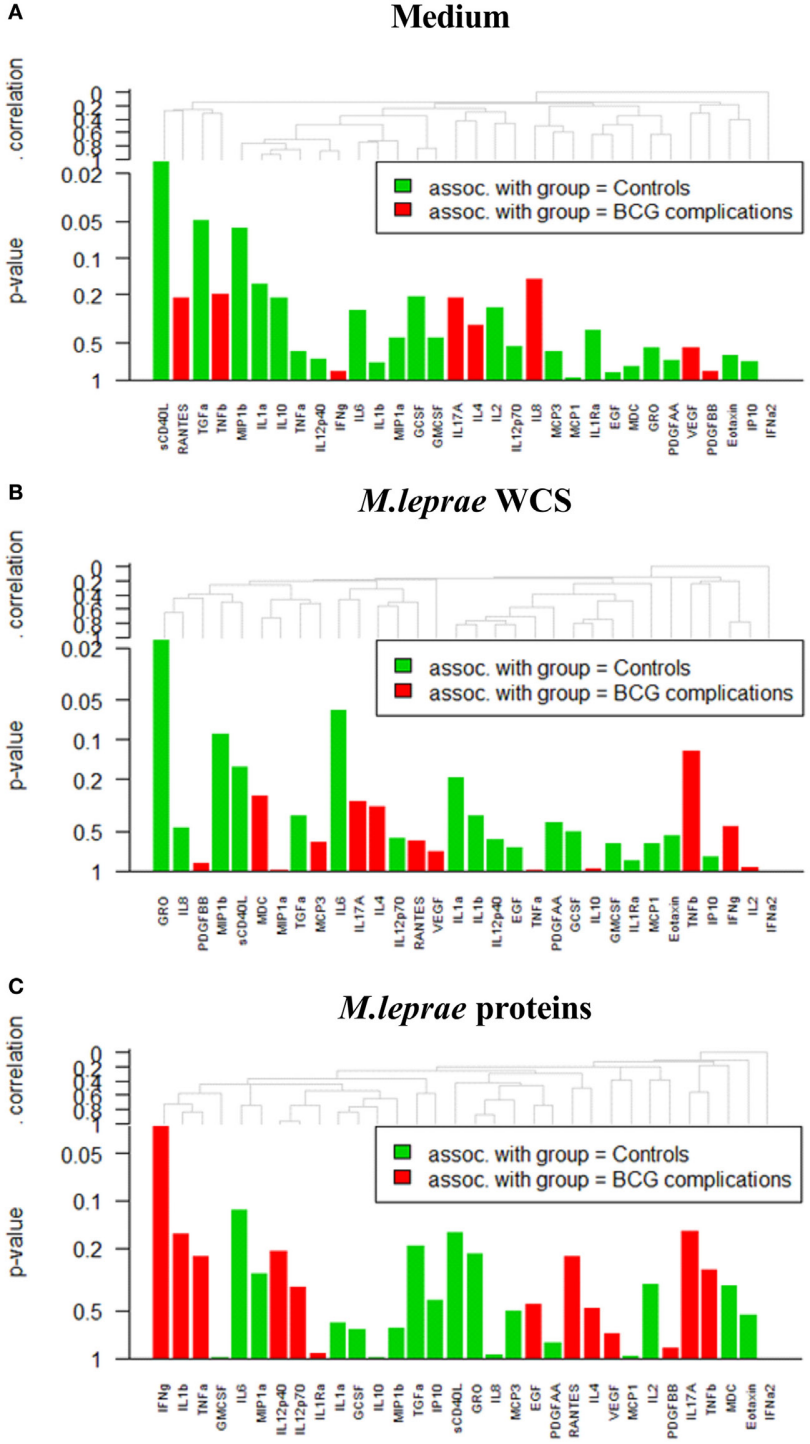
Results of whole-blood assays (WBAs) in contacts with and without BCG complications in **(A)** medium (designated NIL). **(B)** *Mycobacterium leprae* whole cell sonicate (designated WCS). **(C)** ML2478/ML0840 recombinant proteins (designated Mlep) **(C)**.

To assess what type of immune profile (i.e., combinations of cytokines in *M. leprae*-stimulated WBA) is associated with BCG-related complications, a global test (26) was performed on all 32 cytokines stratified by stimulus used in the WBA (Figure 3). This analysis showed that three analytes were significantly different between the two contact groups: decreased levels of sCD40L_NIL_ (soluble cluster of differentiation ligand 40, without stimulation) and GRO_WCS_ (growth-regulated oncogene, in response to *M. leprae* WCS) were significantly associated with occurrence of BCG complications (*p* = 0.03 and 0.013, respectively; Figures 3 and 4). In contrast, increased levels of IFN-γ in response to *M. leprae*-specific proteins (IFN-γ_Mlep_; *p* = 0.012) were observed in individuals developing BCG complications (Figures 3 and 4). Individually these three markers enable a good distinction between contacts with BCG-related complications and those without, showing an AUC of 0.75 for sCD40L and 0.78 for both GRO_WCS_ and IFN-γ_Mlep_ (Figure 3). Using a LDA three additional markers CCL4_NIL_, IL-6_Mlep_, and GCSF_NIL_ that were decreased in individuals with adverse events, were identified, that improved the signature for adverse events. Next, the six analytes were ranked based on their contribution to the discriminant function and sequentially added to the biomarker profile (Table 3) and scored for each individual as positive or negative based on the optimal cutoff. This showed that optimal sensitivity (100%) was observed for the combination of sCD40L_NIL_, IFN-γ_Mlep_, and GRO_WCS_ showing 76% specificity and an AUC of 0.94 (*p* < 0.0001). On the other hand, optimal specificity (100%) was achieved by a five marker profile (sCD40L_NIL_, IFN-γ_Mlep_, GRO_WCS_, CCL4_NIL_, and IL-6_Mlep_), with a sensitivity of 84% and an AUC of 0.96. The cutoff of >3.5 indicates that none of the contacts without complications scores positive for more than 4 out of 5 markers, thereby showing addition of markers improves the specificity. The five marker profile was optimal, as addition of a sixth marker slightly decreased the AUC from 0.96 to 0.93 (Table 3).

**Table 3:** Ability of analytes to distinguish contacts with adverse events in whole blood assays.

	Single markers				Signature			
Step	Analyte	Correlation	Stimulus	*p*-value	AUC	Sens.	Spec.	Cutoff
1	sCD40L	0.086	NIL	0.0262	0.75	85%	54%	<289
2	IFN-γ	0.076	Mlep	0.0124	0.83	62%	92%	>1.5
3	GRO	0.070	WCS	0.0126	0.94	100%	76%	>1.5
4	CCL4	0.066	NIL	0.1254	0.94	92%	85%	>2.5
5	IL-6	−0.055	Mlep	0.2234	0.96	84%	100%	>3.5
6	GCSF	0.043	NIL	0.2428	0.93	85%	92%	>3.5

*Step-by-step addition of analytes ranked by absolute size of correlation within discriminant function. For each step, the analyte that was added to the signature specific for occurrence of BCG vaccination-related adverse events, the absolute size of correlation generated from the linear discriminant analysis, the stimulus, *p*-value (Mann–Whitney *U* test), area under the curve (AUC), and the sensitivity (sens.) and specificity (spec.) based on the optimal cutoff are shown. The following three different stimuli used were: *M. leprae* whole cell sonicate (WCS), ML2478/ML0840 recombinant proteins (Mlep), or without antigen stimulus (NIL)*.

## Discussion

Within a cluster randomized controlled BCG vaccination trial in contacts of leprosy patients in Bangladesh, adverse events were observed in 0.34% of the recipients. These complications consisted primarily (80%) of skin ulcerations and were associated with increased Th1 immunity, inflammation and reduced T-cell regulation in WBA.

Although serious adverse events after BCG vaccination are rare, as many as 95% of BCG recipients have an uncomplicated, local reaction at the site of inoculation, characterized by the appearance of a pustule in combination with pain, swelling, and erythema within 2 to 3 weeks after vaccination. In approximately 70% of the cases, ulceration with drainage occurs at the vaccine site after about 6 weeks, resulting in a lesion of about 5 mm in diameter. Lesions usually heal within 3 months with permanent residual scarring at the vaccination site. Rare local abscesses and ulcers usually occur between 1 and 5 months post-vaccination, but adverse events have also been reported after longer periods of time (28). Lymphadenopathy occurs in the drainage area of the vaccinated site, so is most common in the axilla and sometimes in the cervical lymph nodes (28). Even more uncommon are serious adverse events such as osteitis, osteomyelitis and disseminated infection (19). Disseminated disease following BCG vaccination occurs usually with immunosuppression, such as HIV-infection (16) or genetic immune deficiency (29), which develops in less than one in a million (20).

The incidence of adverse events of 0.34% in this study is comparable with the 0.02 to 5% described in previous studies (13–15, 18, 28). A trial evaluating the incidence of adverse events to primary and booster BCG vaccination in schoolchildren in Salvador, Bahia (Brazil) (14) observed a rate of 0.35 per 1,000 vaccinations, without lethal cases or disseminated infections. Although not statistically significant, adverse events after booster vaccinations were approximately twice the rate compared with primary vaccination with BCG. The median time to onset of complications was 26 days, 12 days shorter than observed in Bangladesh. Similarly, 0.38 out of 1,000 vaccinated individuals developed complications in a study in the Brazilian Amazon (15). In contrast, the risk in the group receiving a revaccination was only >1.05 in the group receiving a first dose, similar to what we found in our Bangladesh study (0.35 versus 0.32%; *p* = 0.68).

The presence of a BCG scar is considered a highly sensitive indicator of the vaccination status as 92% of individuals aged 1–4 months at vaccination, develops a visible scar at 7–12 months of age, which declines to 84% at 4 years (12). When BCG is given to an infant before they are 1 month old, 90% has a scar at 7–12 months of age and 76% has a scar at 4 years. In this study, we used the absence of a BCG scar to designate the lack of previous (childhood) vaccination. However, since 16–24% of BCG vaccinated individuals do not develop a scar, it could be that a larger number of individuals actually received a BCG booster in the MALTALEP trial than is estimated solely based on the presence of a scar.

The development of leprosy after BCG vaccination can be considered an ultimate adverse event. In a previous study (30), we observed an unexpectedly high proportion of new leprosy patients (mainly PB and leprosy type-1 reactions) among apparently healthy HCs of leprosy patients within the first 3 months after BCG vaccination (0.4% of vaccinated contacts). Of these, 43% had a BCG scar before vaccination in the trial. However, it remains unclear whether BCG vaccination merely catalyzes the formation of clinical symptoms in individuals who are bound to develop leprosy, or whether patients would not have developed the disease without this vaccination.

Several recent studies show that BCG alters the innate immune system by trained immunity (5–7). The protective effect against TB induced by neonatal BCG vaccination coincides with protection against heterologous pathogens. This effect is characterized by decreased anti-inflammatory cytokine responses, but increased IL-6 in unstimulated samples (8). In another study, a BCG vaccination-induced increase in IL-6, EGF, and PDGF-AB/BB and decrease in IP-10, IL-2, IL-13, IL-17, GM-CSF, and GRO was observed in response to various non-specific innate immunity stimuli (PAM3Cys, *C. albicans*, and *S. aureus*). Along with this cytokine biomarker signature, increased CD69 expression on NK cells was observed as well (Dockrell, 2017 #483).

T-helper 1 (Th1) host-cellular immunity is generally considered to be key in controlling mycobacterial infections (31). However, clinical presentation of tuberculoid leprosy as well as type-1 (reversal) reactions also coincides with strong *M. leprae*-specific Th1 immunity and high levels of pro-inflammatory cytokines (32).

Despite the apparent homology between the mycobacteria, BCG but not *M. leprae* can stimulate monocytes to initiate a protective type-1 cascade (33). Moreover, *in vitro* exposure of monocytes from healthy donors to *M. leprae* (or *M. leprae* PGL-I) reduced levels of Th1-type cytokines and expression of macrophage type-1 (Mϕ1) cell surface markers (33). In contrast, *ex vivo* stimulation of peripheral blood mononuclear cells (PBMCs) with BCG or purified protein derivative of tuberculin (PPD) from 10-week-old infants in South Africa, who had received neonatal BCG vaccination, showed upregulation of mϕ1-associated genes whereas mϕ2 associated genes were down-regulated (34), indicating BCG-induced protective immunity. Also, in response to *M. leprae*, monocytes from these infants released higher levels of inflammatory cytokines TNF-α and IL-1β compared with monocytes from unvaccinated infants (33). Similarly, cytokine profiles of infants from the United Kingdom receiving BCG vaccination (35) showed that a higher number of IFN-γ^+^ TNF-α^+^ IL-2^+^ multifunctional CD4^+^ T cells were associated with growth inhibition of mycobacteria. Although T-cell activation (HLA-DR^+^CD4^+^ T cells) was a risk factor for TB disease, increased numbers of BCG-specific T cells secreting IFN-γ were detected in BCG vaccinated infants without TB (36). These studies indicate that pro-inflammatory Th1 immunity, although not the only factor, is associated with BCG-induced protection against tuberculosis. Similarly, the Mitsuda reaction measures whether an adequate immune response to an intradermal injection of the heat-killed leprosy bacilli (lepromin) is initiated, as it has a good prognostic value for susceptibility (when negative) or resistance (when positive) to the lepromatous form of leprosy (37). In line with that it was also observed that individuals that showed large local reactogenicity after intradermal BCG administration or lepromin injection are reported to have less risk for leprosy onset (38).

In a BCG vaccination study in 12 tuberculin skin test (TST) and Quantiferon negative, BCG- naïve adults in The Netherlands, local skin reactions varied strongly between individuals (17). It was observed that BCG vaccination induced significant Th1-type immunity (CD4^+^ IFN-γ^+^, IL-2^+^ TNF-α^+^ and CD8^+^ IFN-γ^+^ T cells) in those that presented with high local inflammation responses, with a peak 8-week post-vaccination. Of note is that BCG vaccination significantly increased regulatory CD8^+^ T cells such as CD25^+^ Foxp3^+^ CD39^+^ CD8^+^ T cells as well as CD25^+^ Foxp3^+^ CD39^+^ LAG-3^+^ CCL4^+^ CD8^+^ T cells in low inflammation responders.

Similarly, individuals who developed (skin) complications in Bangladesh also produced higher levels of IFN-γ in response to *M. leprae* antigens around 8 weeks (average 7.9) post-vaccination, although at least 8 out of 13 contacts with BCG complications were not BCG-naïve and the *a priori* chance of exposure to mycobacteria was considerably larger. In contrast to the Dutch cohort, CRP levels were high in both groups and did not differ significantly (Figure S2 in Supplementary Material).

Of note in the current study are the lower levels of sCD40L_NIL_ and GRO_WCS_ that were significantly associated with BCG complications, concomitantly with elevated IFN-γ levels in response to *M. leprae* unique proteins (IFN-γ_Mlep_). GRO (CXCL1) is expressed by macrophages, neutrophils and epithelial cells and has neutrophil chemoattractant activity. Although the role of GRO in leprosy pathology has not been investigated, increase in GRO levels can reduce severity of multiple sclerosis (39). This neuroprotective role for CXCL1 could well be consistent with the onset of complications upon its reduction after *M. leprae* WCS stimulation as observed in our study. Moreover, in UK-born, BCG-vaccinated infants the levels of GRO in response to non-specific innate immunity stimuli were suppressed as well, in line with our finding in Bangladesh (5).

Recently, it was shown that higher levels of sCD40L present in serum of patients with Behçet’s disease caused a strong stimulus on the production of reactive oxygen species (40). Thus, the reduction in sCD40L observed in contacts with complications could indicate a weaker ability to combat BCG bacilli locally leading to tissue destruction at the vaccination site.

Besides induction of activated T cells, BCG vaccination can also induce regulatory T cells (Tregs), in particular CD8^+^ T cells which dampen the inflammatory response to mycobacteria (41, 42) and lead to inadequate killing of mycobacteria (43). Likewise, Tregs have been isolated from lepromatous leprosy patients, who in contrast to tuberculoid patients display reduced Th1 immunity and capacity to kill *M. leprae* bacteria (44). The breakdown of T-cell regulation, in favor of inflammation, underlies the etiology of tissue damage in tuberculoid leprosy and leprosy reactions (45).

Regulatory T cells can suppress Th1 cells through the secretion of CC chemokine ligand 4 (CCL4) (42). In this study, a reduction in CCL4 (although not significant) could indicate decreased T-cell regulation in individuals with complications, causing a shift in the equilibrium toward excessive Th1-type immunity with corresponding inflammation at the BCG vaccination site. However, further research will be required to identify in detail the cellular subtypes involved. Furthermore, the leprosy contacts with high inflammatory responses after BCG vaccination could therefore also be more likely to develop tuberculoid leprosy. In line with this hypothesis are the two cases out of the 50 contacts in this study with BCG complications, who developed border line tuberculoid leprosy (BT).

## Conclusion

The rate of documented adverse events after BCG vaccination in the studied Bangladesh cohort of leprosy patients’ contacts was low (0.34%), and comparable to studies in other countries.

Contacts with BCG complications showed increased *M. leprae*-specific Th1-type immunity but a tendency of reduced T-cell regulation in WBA with corresponding inflammation at the BCG vaccination site indicating improved protection against*M. leprae*. In addition, these individuals may also be at a higher risk of developing tuberculoid leprosy after *M. leprae* infection.

## Ethics Statement

The MALTALEP trial is performed according to standard Good Clinical Practice (GCP) guidelines. Participants were informed about the study objectives, the samples, and their right to refuse to take part in or withdraw from the study without consequences for their treatment. Written informed consent was obtained before enrolment. For illiterate people, a thumb print was taken, and for minors under 16 years of age, the guardian’s additional consent was taken. All patients received treatment according to national guidelines. Participants were informed about the potential adverse events of the trial, that free consultation and treatment would be offered in case of adverse events and requested to report any suspected adverse events to the responsible field worker. Ethical approval of the study-protocol was obtained through the National Research Ethics Committee (Bangladesh Medical Research Council; Protocol no. BMRC/NREC/2010-2013/1534).

## Author Contributions

This research project was designed by the authors JR and AG. Patients were enrolled and a clinical diagnosis was performed and registered by the field staff under supervision of KA and RR. The laboratory testing was done by AH, SE, and LW. The data were analyzed by RR, AH, JR, and AG. The paper was written by RR and AG. All authors agreed with manuscript results and conclusions.

## Conflict of Interest Statement

The authors declare that the research was conducted in the absence of any commercial or financial relationships that could be construed as a potential conflict of interest.

## Footnotes

^1^http://www.who.int/immunization/monitoring_surveillance/data/bgd.pdf (Accessed: March 19, 2018).

^2^www.ich.org (Accessed: March 19, 2018).

^3^http://www.beiresources.org/TBVTRMResearchMaterials/tabid/1431/Default.aspx (Accessed: March 19, 2018).

^4^http://www.graphpad.com (Accessed: March 19, 2018).

^5^http://www.spss.com.hk (Accessed: March 19, 2018).

^6^http://www.R-project.org (Accessed: March 19, 2018).
