# Supplementary material for: BCG and Adverse Events in the Context of Leprosy
Source: Front Immunol. 2018 Apr 4;9:629. doi: 10.3389/fimmu.2018.00629 (PMC5893643; doi:10.3389/fimmu.2018.00629)
Supplement: Supplementary file 1 [file image_1.PDF]

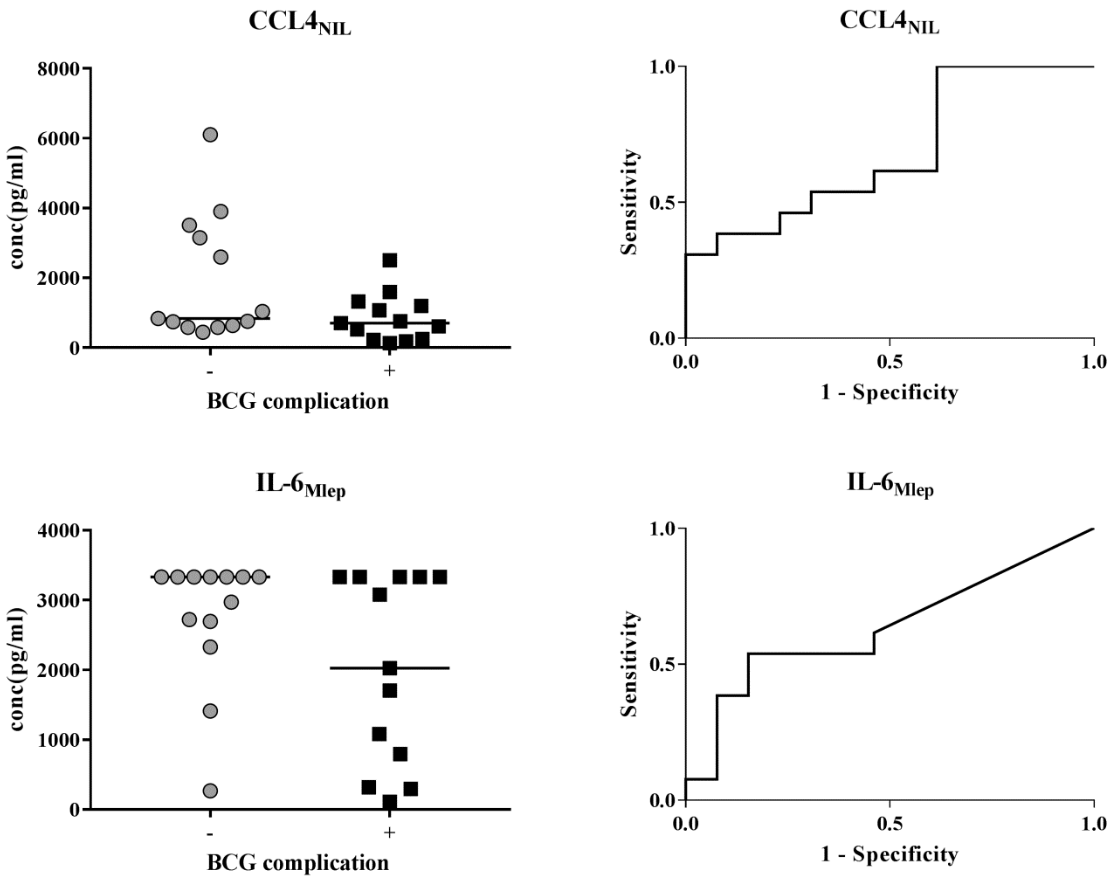

Supplementary Figure 1

Cytokine concentrations in 24h whole-blood assays (WBA) without (NIL) or with stimulation with M. leprae unique proteins (Mlep) or M. leprae whole cell sonicate (WCS) in contacts with and without BCG complications (left panels). Receiver operating characteristic curves (ROC) were computed and the area under the curve (AUC) is indicated for each analyte (right panels). The limits of detections for CCL4 were 1.3 to 10,000 and for IL-6 were 1.5 to 3,333.
