# Supplementary material for: BCG and Adverse Events in the Context of Leprosy
Source: Front Immunol. 2018 Apr 4;9:629. doi: 10.3389/fimmu.2018.00629 (PMC5893643; doi:10.3389/fimmu.2018.00629)
Supplement: Supplementary file 2 [file image_2.PDF]

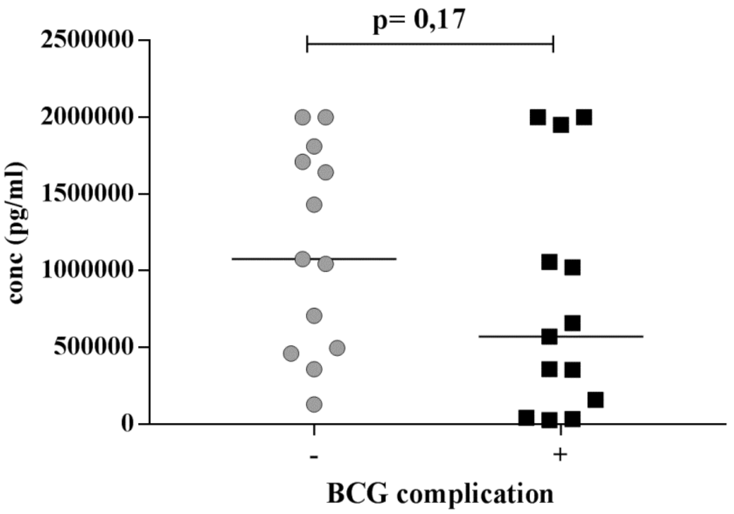

Supplementary Figure 2

CRP concentrations determined by ELISA in plasma samples of contacts with and without BCG complications.
