# Supplementary material for: BCG and Adverse Events in the Context of Leprosy
Source: Front Immunol. 2018 Apr 4;9:629. doi: 10.3389/fimmu.2018.00629 (PMC5893643; doi:10.3389/fimmu.2018.00629)
Supplement: Supplementary file 3 [file table_1.PDF]

Supplementary Table S1: *Characteristics of contacts and their complication after BCG*

Nr.	Age	Sex	Genetically related to index case***	Contact level****	BCG scar	Leprosy at FU1	Time between BCG and onset of complication (in weeks)	Ulcer > 10 mm	Lymph-adenopathy	Keloid	Other	Treated?
1	30	F	Y	H	N	N	2	Y	N	N	N	Y
2	50	F	N	H	Y	N	8	Y?	N	N	N	Y
3	72	M	Y	N	N	N	8	Y	N	N	N	Y
4	11	F	Y	H	N	Y	8	N	N	Y	N	Y
5	40	F	N	N	Y	N	4	N	N	Y	Y	Y
6	37	F	N	N	N	N	2	Y	N	N	N	Y
7	40	M	Y	N	Y	N	8	Y	N	N	N	Y
8	75	M	Y	N	N	N	4	Y	N	N	N	Y
9	13	F	Y	H	Y	N	1	Y	N	N	N	N
10	18	M	N+	N	Y	N	1	N	N	Y	N	Y
11	48	M	Y	H	Y	N	20	N	Y	N	N	Y
12	25	M	Y	H	N	N	1	Y	N	N	N	N
13	45	F	Y	H	Y	N	1	Y	N	N	N	Y
14	7	M	Y	H	Y	N	8	Y	N	N	N	Y
15	40	F	N	N	N	N	2	Y	N	N	N	Y
16	10	M	Y	H	Y	N	6	Y	N	N	N	Y
17	7	F	Y	H	Y	N	6	Y	N	N	N	Y
18	8	F	Y	H	Y	N	4	Y	N	N	N	Y
19	15	M	N	N	Y	N	8	Y	N	N	N	Y
20	14	F	Y	H	Y	N	20	N	N	N	Y	Y
21	60	M	N+	N	N	N	1	Y	N	N	N	Y
22	10	M	N+	N	Y	N	4	Y	N	N	N	Y
23	14	M	N+	N	Y	Y	1	Y	Y	N	N	Y
24	6	F	N+	N	Y	N	3	Y	N	N	N	Y
25	35	F	N	N	N	N	1	Y	N	N	N	Y
26	30	F	N	H	N	N	8	Y	N	N	N	Y
27	38	F	N	H	N	N	2	Y	N	N	N	Y
28	7	M	N+	N	Y	N	8	Y	N	N	N	Y
29	65	M	N	H	N	nd FU1*	4	Y	N	N	N	Y
30	45	M	N	H	N	N	2	Y	Y	N	N	Y
31	26	M	N+	N	N	N	4	Y	N	N	N	N
32	18	M	N+	N	Y	N	52	Y	N	Y	N	N
33	80	F	Y	N	N	N	1	Y	N	N	N	Y
34	35	F	N	N	Y	N	7	Y	N	N	N	Y
35	6	F	Y	H	N	N	16	Y	N	N	N	Y
36	6	F	N+	H	Y	N	6	Y	N	N	N	Y
37	50	F	Y	H	Y	N	3	Y	N	N	N	Y
38	50	M	N+	N	N	N	1	Y	Y	N	Y	Y
39	6	F	N	N	Y	N	10	N	N	Y (bs**)	Y	Y
40	10	M	N	N	Y	N	8	Y	N	N	N	Y
41	6	M	N+	H	Y	N	2	Y	N	N	N	Y
42	14	M	Y	H	Y	N	10	N	N	Y (bs**)	N	N
43	10	M	Y	H	Y	N	10	N	N	Y (bs**)	N	N
44	67	F	N	H	N	N	11	Y	N	N	N	Y
45	60	F	Y	H	N	N	8	Y	N	N	N	Y
46	28	F	Y	H	Y	N	1	Y	Y	N	N	?
47	60	F	N+	H	N	N	1	Y	N	N	N	?
48	6	M	N+	N	Y	N	8	Y	N	N	N	Y
49	9	F	N+	N	Y	N	6	N	N	Y	N	Y
50	40	F	N	H	Y	N	1	Y	N	N	N	Y

*nd FU1: not done follow-up 1 at 8-12 weeks
**bs: big scar >10 mm
***Blood relation to index: child (son/daughter), parent (father/mother), brother or sister;
[+]Other relative: unclear if blood related or not
****Contact level: H= household contact; sharing either the same roof or kitchen, or both;
N= neighbour living next door to patient`s house
